# Associations of Psychological Distress, Cognitive Function, and Physical Activity with Daily Functioning and Quality of Life Across Disability Levels in Multiple Sclerosis

**DOI:** 10.3390/medicina62020316

**Published:** 2026-02-03

**Authors:** Saad A. Alhammad, Hamad T. Aldubayan, Majed S. Albalawi, Alaa A. Mutawam, Sami S. Alabdulwahab

**Affiliations:** 1 Rehabilitation Sciences Department, Faculty of Applied Medical Sciences, King Saud University, Riyadh 11411, Saudi Arabia; 2 Rehabilitation Department, Dammam Medical Complex, Al Khobar 31451, Saudi Arabia; 3 Physical Therapy Department, Prince Fahad bin Sultan Hospital, Tabuk 47311, Saudi Arabia; 4 Rehabilitation Department, Abu Areesh General Hospital, Jazan 45911, Saudi Arabia

**Keywords:** multiple sclerosis, psychological distress, cognitive function, physical activity, quality of life

## Abstract

*Background and Objectives*: Multiple sclerosis (MS) is a chronic neurological disorder causing physical, cognitive, and psychological impairments that affect daily functioning and quality of life (QoL). Psychological distress, cognitive deficits, and reduced physical activity often co-occur, yet their associations with QoL across disability levels are unclear. This study examined these relationships in people with relapsing–remitting MS, stratified by disability severity. *Materials and Methods*: This cross-sectional study included 149 adults with RRMS. Disability severity was classified as mild, moderate, or severe using the Patient-Determined Disease Steps (PDDS) scale. Psychological distress was assessed using the Depression, Anxiety, and Stress Scale–21 (DASS-21), cognitive function using the Montreal Cognitive Assessment (MoCA), and physical activity using the International Physical Activity Questionnaire (IPAQ). QoL was evaluated using the Multiple Sclerosis International Quality of Life (MusiQoL) questionnaire. Stratified comparative analyses were conducted to examine differences in overall and domain-specific QoL according to levels of psychological distress, cognitive function, and physical activity within disability categories. *Results*: In participants with mild and moderate disability, higher levels of depression, anxiety, and stress were associated with lower QoL scores, particularly in domains related to activities of daily living, psychological well-being, and symptoms. Higher cognitive function and greater physical activity were associated with more favorable QoL across several domains. In those with severe disability, associations between psychological distress and QoL were less consistent, although stress remained associated with selected QoL domains. *Conclusions*: Psychological distress, cognitive function, and physical activity show distinct patterns of association with daily functioning and QoL across disability levels in RRMS. Although causal inferences cannot be drawn from this cross-sectional design, disability-stratified analyses provide clinically relevant insights into how these factors co-vary with QoL at different stages of disease severity.

## 1. Introduction

Multiple sclerosis (MS) is a chronic, immune-mediated neurological disorder characterized by inflammatory demyelination and neurodegeneration of the central nervous system. It typically begins in early adulthood and is one of the most common causes of non-traumatic disability among young adults worldwide [1]. The disease course is heterogeneous. Relapsing–remitting MS (RRMS) accounts for approximately 85% of initial diagnoses, followed by secondary-progressive, primary-progressive, and the less common progressive-relapsing forms [2]. Regardless of disease subtype, MS imposes a substantial personal and societal burden. It often affects individuals during their most productive years and is associated with reduced participation in work and social roles, as well as poorer quality of life (QoL) [3].

Individuals with MS present with a wide range of physical, cognitive, and psychological symptoms. Common manifestations include motor impairment, fatigue, spasticity, sensory disturbances, pain, cognitive dysfunction, and emotional distress [4]. As disability accumulates, these symptoms frequently co-occur. Their combined presence is associated with increasing difficulty in daily activities and social functioning [5]. Cognitive impairment affects up to 60% of people with MS, including those in the early stages of the disease. Deficits are most often observed in attention, memory, processing speed, executive function, and verbal fluency [6,7]. Psychological symptoms are also prevalent. Depression and anxiety are frequently reported, with depression affecting up to 50–60% of individuals over the disease course and consistently associated with poorer QoL [8].

Physical symptoms such as weakness, balance impairment, pain, spasticity, and fatigue further shape the lived experience of MS [9,10]. Fatigue, in particular, is among the most disabling symptoms and is closely associated with reduced participation in physical activity and daily routines. Importantly, impairments across physical, cognitive, and emotional domains rarely occur in isolation. Instead, they tend to cluster, and their co-occurrence is associated with lower QoL [11,12]. Consequently, QoL has become a central outcome in MS research and clinical care. It reflects patients’ perceptions of physical, psychological, and social well-being and complements traditional clinical measures of disease severity [13]. Both generic and MS-specific patient-reported outcome measures, including the Multiple Sclerosis International Quality of Life (MusiQoL) questionnaire, are widely used to capture these multidimensional experiences [14].

The experience and reporting of MS symptoms and QoL may also vary across cultural and regional contexts. Evidence from Saudi Arabia suggests that cultural factors, such as family support, social expectations, and engagement in community or religious activities, influence both the experience of MS and self-reported QoL [15,16]. For instance, participation in culturally valued physical or social activities may buffer the impact of fatigue and psychological distress, underscoring the importance of considering cultural context when evaluating QoL in MS. Physical activity is therefore widely recommended as a key component of MS rehabilitation, including in regional clinical practice guidelines [17]. Despite growing interest in patient-reported outcomes, relatively few studies have examined how psychological distress, cognitive function, and physical activity are simultaneously associated with QoL across different levels of disability. In particular, limited attention has been given to whether these associations differ between individuals with mild, moderate, and severe disability [6,8,18].

Given these limitations, a cross-sectional, disability-stratified analyses offer a useful approach for describing patterns of association between symptoms and QoL at clinically meaningful stages of disease severity. Although causal inferences cannot be drawn from such designs, they provide important insights into how symptom burden and functional limitations co-vary within distinct disability groups. These insights can inform hypothesis generation and support more tailored assessment and supportive care strategies.

Accordingly, this study aimed to examine the associations between cognitive function, physical activity, psychological distress (anxiety, stress, and depression), and QoL among people with MS, stratified by disability severity (mild, moderate, and severe). We hypothesize that higher disability will be associated with greater symptom burden, and that psychological, cognitive, and physical factors will differentially relate to QoL across disability levels, reflecting the multifactorial determinants of functioning in RRMS.

## 2. Materials and Methods

### 2.1. Study Design, Setting, and Patients

This cross-sectional study included 149 patients with RRMS living in Saudi Arabia. Participants were recruited through random sampling from the registries of the Saaed Assembly (Riyadh) and the Arfa Organization (Dammam). Of 250 eligible patients with RRMS, we randomly selected 210 using a computer-generated random-number list. Of those selected, 149 agreed and volunteered to participate in the study. The 149 patients were stratified into three groups based on PDDS severity levels (mild, moderate, and severe).

Participants were Saudi males and females aged 20–50 years with a confirmed diagnosis of relapsing–remitting MS. To minimize the confounding effects of recent disease activity on cognitive, psychological, and QoL measures, only patients whose last relapse occurred ≥2 years prior were included. This period was chosen because previous studies indicate that physical, cognitive, and psychological function tend to stabilize after two years of remission, reducing short-term variability associated with relapse.

Exclusion criteria were (1) use of medications known to affect cognition or psychometric performance; (2) diagnosis of additional chronic diseases influencing QoL; (3) history of neurological or psychiatric disorders unrelated to MS; or (4) pregnancy.

### 2.2. Sample Size Estimation

The required sample size was estimated using G*Power software (version 3.1) to detect a small-to-moderate effect size (f = 0.25) with α = 0.05 and 80% power. This calculation accounted for stratification across three disability levels, ensuring sufficient power for subgroup analyses of QoL, cognitive function, and physical activity within each disability stratum. Based on these assumptions, a minimum of 149 participants was required, ensuring sufficient statistical power for inferential analyses [19].

### 2.3. Outcome Measures

Five validated instruments were used to assess disability, cognition, physical activity, emotional status, and QoL.

#### 2.3.1. Patient-Determined Disease Steps (PDDS)

A nine-item self-reported scale measuring functional disability in MS. Scores range from 0 (normal) to 8 (bedridden). It has demonstrated reliability and validity in both English and Arabic [20,21]. For this study, disability severity was classified as mild (0–3), moderate (4–6), and severe (≥7).

#### 2.3.2. Montreal Cognitive Assessment (MoCA)

A brief cognitive screening tool evaluating attention, executive function, memory, language, visuospatial skills, and abstraction. Scores range from 0–30, with <26 indicating cognitive impairment. The MoCA has been validated in Arabic and is widely used in MS populations [22].

#### 2.3.3. International Physical Activity Questionnaire (IPAQ)

A standardized tool measuring physical activity levels over the previous 7 days, including walking, moderate, and vigorous activities, as well as sedentary behavior. Results are reported in metabolic equivalent task (MET)-minutes/week [23,24].

#### 2.3.4. Depression, Anxiety, and Stress Scale (DASS-21)

A 21-item self-report instrument assessing depression, anxiety, and stress on three subscales (7 items each). Scores are rated on a 4-point Likert scale, with higher scores indicating greater symptom severity. The Arabic version is validated and reliable [25].

#### 2.3.5. Multiple Sclerosis International Quality of Life (MusiQoL)

A 31-item self-reported measure covering nine domains of QoL, including daily activities (ADL), psychological well-being, symptoms, relationships, and social participation. Higher scores indicate better QoL. The Arabic version is validated [26,27].

### 2.4. Procedures

Eligible patients were contacted through the registries after obtaining ethical approval and provided written informed consent before participation. Data collection was conducted in person, with questionnaires administered sequentially (PDDS, MoCA, IPAQ, DASS-21, MusiQoL). Each session lasted approximately 45–60 min. Participants were allowed breaks to reduce fatigue.

Ethical approval from the Institutional Review Board at King Saud University was granted before commencing data collection. All patients received an explanation of the study’s nature and procedure. They were informed that their participation is voluntary and that they can withdraw from the study at any time. Afterward, they were asked to sign a consent form before participation. Then, they completed five questionnaires face to face, starting with the PDDS, MoCA, IPAQ, DASS-21, and MusiQoL, respectively. The questionnaires were filled exactly as described in the previous sections. If they felt tired, the rest of the questionnaires were completed later that day. Then, based on the PDDS level of disability, patients were grouped into three groups: mild, moderate, and severe.

### 2.5. Statistical Analysis

All statistical analyses were performed using IBM SPSS Statistics for Windows, version 26.0 (IBM Corp., Armonk, NY, USA). Missing data were handled using case-wise (listwise) deletion. Descriptive statistics were used to summarize participant characteristics and study variables. Continuous variables are presented as means with standard deviations (SDs), whereas categorical variables are reported as frequencies and percentages. The significance between groups was examined using one-way analysis of variance (ANOVA) with Tukey’s post hoc tests for the continuous variable of age, while a Chi-square test was used for categorical variables, such as education, gender, anxiety, stress, and depression. The distributional assumptions of continuous variables were assessed using the Shapiro–Wilk test.

Comparisons of continuous outcomes, including QoL, cognitive function, and physical activity, across disability severity groups were conducted using one-way ANOVA with Tukey’s post hoc tests applied for pairwise comparisons when appropriate. In addition, stratified analyses were performed within each disability category (mild, moderate, and severe). Within each stratum, differences in MusiQoL domain scores according to cognitive function (low vs. normal) were examined using independent samples t-tests, while differences according to physical activity level (low or no activity, moderate activity, and high activity) were assessed using one-way ANOVA.

All statistical tests were two-sided, and a *p* value of less than 0.05 was considered statistically significant.

## 3. Results

### 3.1. Participant Characteristics

Basic characteristics of the study sample are summarized in Table 1. Participants with severe disability were older (mean 40.8 ± 4.8 years) than those with moderate (34.6 ± 4.4 years) or mild disability (32.1 ± 5.7 years). Most participants had a university-level education, although a higher proportion of severe cases reported only a high school education (26.7%) compared with moderate (13%) and mild cases (6.2%). Men were more frequently represented in the severe group (73.3%) than in the moderate (62.3%) or mild (53.8%) groups.

Psychological symptoms varied across disability levels. Moderate anxiety was most common in severe cases (53.3%), whereas mild anxiety predominated in the moderate group (44.9%). Severe or extremely severe anxiety was uncommon. Stress levels showed a clear gradient: normal stress was reported by 64.6% of mild cases, 31.9% of moderate cases, and only 6.7% of severe cases. Severe stress was more frequent among severe cases (33.3%) than in moderate (5.8%) or mild (1.5%) groups. Depression also differed by disability: over half of severe cases reported mild depression (53.3%), while moderate depression predominated in the moderate group (52.2%). Severe and extremely severe depression were rare across all groups.

### 3.2. Functional Outcomes

**Figure 1:**
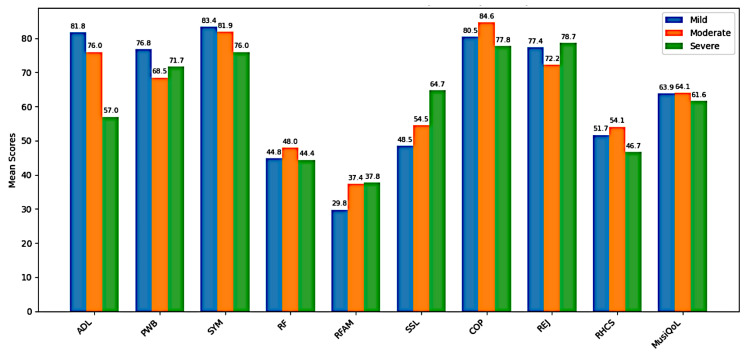
ADL and MusiQoL domain scores across disability levels in multiple sclerosis. Abbreviations: ADL, activity of daily living; PWB, physical well-being; SYM, symptoms; RF, relationship with friends; RFAM, relationships with family; SSL, sentimental and sexual life; COP, coping; REJ, rejection; RHCS, relationship with healthcare system; MusiQoL, musiquality of life total score. Note: Disability severity was categorized as mild (0–3), moderate (4–6), and severe (7 or higher) based on the PDDS.

Functional outcomes showed progressive decline with increasing disability. ADL scores decreased from mild (81.8 ± 9.8) to severe cases (57.0 ± 9.6). Psychological well-being (PWB) and symptom burden (SYM) were slightly lower in moderate and severe groups compared with mild. Scores for relationships with friends (RF) and family (RFAM) were generally similar across groups, though RFAM was higher in moderate and severe disability. Social support (SSL) increased with disability severity, peaking in the severe group (64.7 ± 13.6). Coping (COP) was highest in the moderate group (84.6 ± 12.8), while rejection (REJ) remained consistent across groups. Relationship with healthcare system (RHCS) scores were slightly lower among severe cases (46.7 ± 9.0). Overall MusiQoL scores were comparable across groups, with only a modest decline in severe disability (61.6 ± 3.0) relative to mild (63.9 ± 5.6) and moderate (64.1 ± 5.2) groups (Figure 1).

### 3.3. Distribution of Cognitive Function and Physical Activity by Disability Severity

**Figure 2:**
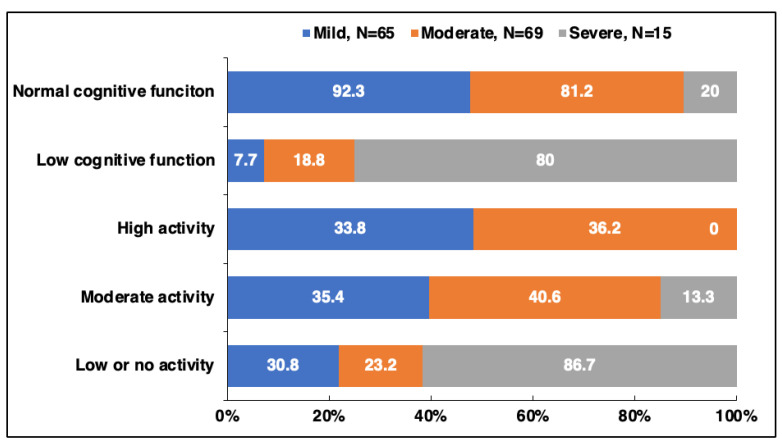
Distribution of cognitive function and physical activity across disability severity in patients with RRMS. Note: Disability severity was categorized as mild (0–3), moderate (4–6), and severe (7 or higher) based on the PDDS. The study included 65 participants in the mild group, 69 in the moderate group, and 15 in the severe group.

Patterns of cognitive function and physical activity varied across disability levels (Figure 2). Among patients with mild disability, most (92.3%) demonstrated normal cognitive function, with only 7.7% showing low function. In contrast, 18.8% of moderate and 80% of severe cases exhibited low cognitive function, indicating a clear association between greater disability and cognitive decline.

Physical activity levels also decreased with increasing disability. High activity was reported by 33.8% of mild and 36.2% of moderate cases but by none of the severe cases. Moderate activity was most common in the moderate group (40.6%), compared with 35.4% in mild and 13.3% in severe cases. Low or no activity was reported in 30.8% of mild and 23.2% of moderate cases, but it was most prevalent among severe cases (86.7%).

### 3.4. Associations Between Cognitive Function, Physical Activity, and QoL Domains by Disability Severity

The associations of cognitive function (low vs. normal) and physical activity (low, moderate, high) with QoL domains (MusiQoL subscales) and ADL across disability groups are summarized in Table 2.

#### 3.4.1. Mild Disability

Participants with normal cognitive function reported higher ADL scores than those with low cognition (82.8 ± 9.2 vs. 69.5 ± 8.3, *p* = 0.003). No other domains differed significantly by cognitive function. Regarding physical activity, significant associations were observed for psychological well-being (PWB, *p* = 0.029), rejection (REJ, *p* < 0.001), and relationship with healthcare system (RHCS, *p* < 0.001). Participants with higher activity levels reported better PWB, lower REJ, and higher RHCS compared with those with low or moderate activity.

#### 3.4.2. Moderate Disability

Normal cognitive function was associated with higher ADL scores than low cognition (77.7 ± 8.9 vs. 68.6 ± 10.3, *p* = 0.002). Physical activity was significantly associated with symptom burden (SYM, *p* = 0.013), relationship with friends (RF, *p* = 0.025), and relationship with family (RFAM, *p* = 0.041). Higher activity levels were associated with fewer symptoms and improved social functioning. Overall MusiQoL scores did not differ significantly by cognitive function or physical activity.

#### 3.4.3. Severe Disability

Cognitive function showed no significant associations with ADL or most QoL domains, except for sentimental and sexual life (SSL), which was higher among participants with low cognition compared with normal cognition (68.3 ± 11.9 vs. 50.0 ± 10.0, *p* = 0.030). Physical activity was not significantly associated with ADL or QoL in this group.

### 3.5. Association Between Psychological Distress and QoL Domains by Disability Severity

The associations between psychological distress (anxiety, stress, depression) and QoL are presented in Table 3, stratified by disability.

#### 3.5.1. Mild Disability

Higher severity of anxiety, stress, and depression was generally associated with lower ADL and PWB scores (all *p* ≤ 0.009). Symptom burden (SYM) increased with higher anxiety (*p* = 0.001) and depression (*p* = 0.003), but not stress (*p* = 0.368). Anxiety also influenced relationships with friends (RF, *p* = 0.028) and the healthcare system (RHCS, *p* = 0.026). Coping (COP) decreased with higher anxiety (*p* = 0.030), while REJ scores were higher among participants with more severe stress (*p* = 0.011) and depression (*p* = 0.004).

#### 3.5.2. Moderate Disability

Greater severity of anxiety, stress, and depression was associated with lower ADL and PWB scores (all *p* ≤ 0.046). Symptom burden increased with higher anxiety (*p* < 0.001) and stress (*p* = 0.032), while depression showed no significant effect (*p* = 0.269). Anxiety was associated with RF (*p* < 0.001), RFAM (*p* < 0.001), and COP (*p* = 0.003). Stress and depression also affected RFAM and COP (all *p* ≤ 0.001). Overall MusiQoL scores declined with increasing anxiety (*p* = 0.006), stress (*p* = 0.002), and depression (*p* = 0.008), while SSL, REJ, and some other domains were largely unaffected.

#### 3.5.3. Severe Disability

Anxiety severity was not significantly associated with ADL, PWB, QoL subscales, or overall MusiQoL scores (all *p* > 0.05). Higher stress levels were associated with poorer ADL (*p* = 0.013) and lower PWB (*p* = 0.033), as well as differences in SSL (*p* = 0.016) and RHCS (*p* = 0.016). Other domains, including SYM, RF, RFAM, COP, and REJ, did not vary significantly by stress. Depression severity was not significantly associated with any ADL or QoL outcomes in this group.

### 3.6. Heatmap of Quality of Life and Functioning Across Psychological Distress and Disability Levels

**Figure 3:**
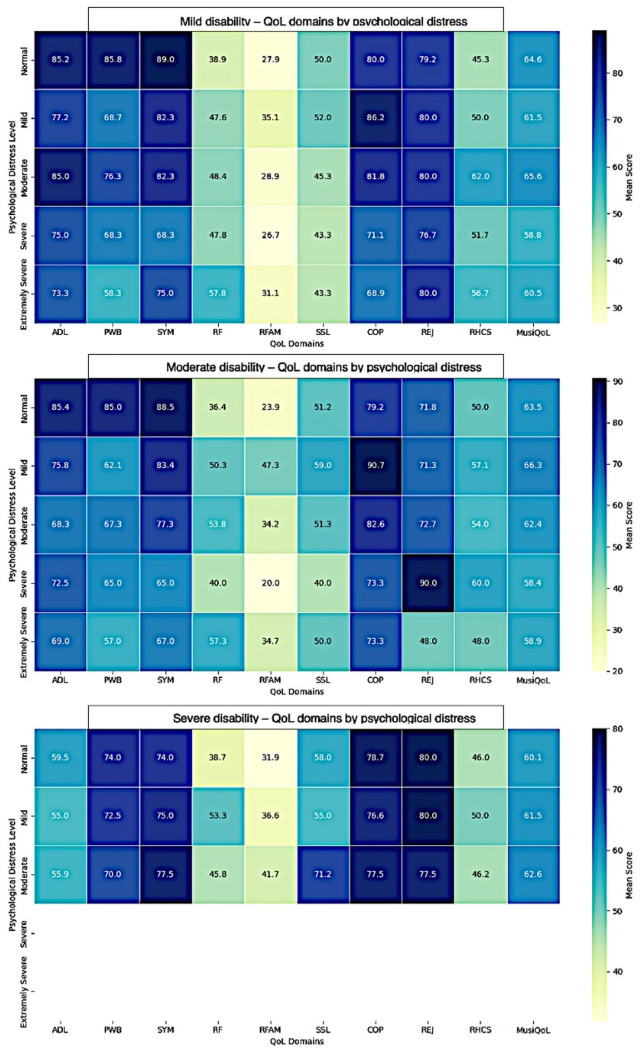
Heatmaps of quality of life and daily functioning across psychological distress levels in multiple sclerosis patients. Note: The heatmaps visually highlight trends in domain scores and the relative impact of psychological distress, with darker colors indicating higher scores. Notably, in severe disability, data sparsity may influence the observed patterns.

Figure 3 presents heatmaps of mean scores for quality of life and daily functioning domains (ADL, PWB, SYM, RF, RFAM, SSL, COP, REJ, RHCS, MusiQoL) across anxiety, stress, and depression categories, stratified by disability severity. In patients with mild disability, lower psychological distress was generally associated with higher ADL, PWB, SYM, and COP scores, whereas higher levels of anxiety, stress, or depression corresponded with lower scores across most domains. For moderate disability, similar patterns were observed, although some domains (e.g., RFAM, SSL) showed less pronounced differences. In the severe disability group, variations across distress levels were minimal, likely reflecting either attenuation of psychological impact or the limited subgroup size. Overall, the heatmaps provide a clear visual summary of the relationships between psychological distress and functional and quality of life outcomes, highlighting stronger associations in mild-to-moderate disability and relative stability in severe disability.

## 4. Discussion

This study examined associations between psychological distress, cognitive function, and physical activity with daily functioning and QoL in MS patients across mild, moderate, and severe disability levels. We found that anxiety, stress, and depression were associated with lower QoL, particularly in domains of ADL, PWB, SYM, COP, RF, and RFAM among patients with mild-to-moderate disability. In patients with severe disability, only stress showed an association with ADL and PWB, whereas anxiety and depression were largely unrelated to QoL outcomes. These results suggest that psychological distress is more strongly linked to functional and psychological outcomes in patients with lower disability, while its relative influence diminishes as physical disability increases.

Participants with more severe disability were significantly older, indicating a progressive increase in disability with age. Educational attainment also differed across groups, with a lower proportion of university-educated individuals in the severe disability group, suggesting potential social or functional disadvantages accompanying advanced disability. Gender distribution did not differ significantly, indicating that disability severity was not sex-dependent in this cohort.

Psychological distress increased markedly with disability severity. Anxiety, stress, and depression showed statistically significant associations, with moderate-to-severe symptom levels more prevalent among participants with severe disability. Notably, stress exhibited the strongest gradient, with a substantial shift from normal levels in mild disability to severe levels in the severe disability group. Similarly, depressive symptoms progressed from predominantly normal or mild levels in the mild group to higher proportions of moderate and severe depression in the moderate and severe groups, respectively.

Overall, these findings highlight the close interplay between advancing disability, aging, and psychological burden, underscoring the importance of integrating mental health assessment and support into the clinical management of individuals across all stages of disability, particularly those with moderate to severe impairment. Although the present study offers valuable insights from a Saudi Arabian MS cohort, cultural, social, and healthcare system factors may shape how patients perceive and report QoL, social support, and psychological distress. Cultural values influence individuals’ expectations, social roles, coping strategies, and interpretations of symptoms, outcomes that are not universally uniform across populations. QoL is known to be a subjective and culturally constructed concept, with cultural background affecting how people appraise well-being, social connectedness, and functional limitations [28]. Qualitative research in MS further suggests that sociocultural norms and beliefs can influence patients’ lived experiences, including stress, coping, and stigma [29].

In the Saudi context, family expectations and societal roles may uniquely influence psychological responses and reported outcomes, and healthcare access patterns may differ from those in Western settings [30]. Therefore, while the observed patterns likely reflect real associations in this cohort, caution is warranted when generalizing the findings to non-Middle Eastern or non-Arab populations. Future research in diverse cultural settings is needed to determine whether these associations are consistent internationally and to inform culturally tailored interventions.

Our findings are consistent with previous studies showing that emotional distress is associated with poorer functioning and QoL in MS. Depression and anxiety are prevalent in MS and are linked to reduced QoL independent of physical disability [8]. Cognitive impairment, particularly in memory, attention, and executive function, is also associated with reduced independence and participation in daily activities [6,7]. Physical symptoms, including fatigue and weakness, limit daily functioning and reduce engagement in physical and social activities [10,16]. The attenuated association of psychological distress in severe disability may reflect a ceiling effect, where physical limitations dominate QoL and reduce the observable impact of emotional symptoms, as suggested by prior research [6].

### 4.1. Strengths and Limitations

Key strengths of this study include the use of standardized, validated instruments (DASS-21, MoCA, IPAQ, MusiQoL) to capture multiple domains of ADL and QoL, and stratification by disability level to identify differential effects [31,32]. The inclusion of both cognitive and physical activity measures alongside psychological distress provides a holistic view of factors influencing QoL [33].

A potential limitation of the study is that it categorized MS patients into mild, moderate, and severe disability, a common approach in previous research, which may limit the novelty of the analytical strategy [34,35]. The small sample size, particularly in the severe disability group, precluded multivariable modeling or testing interactions between disability and key determinants, restricting the findings to descriptive and associative observations. Future studies with larger cohorts are warranted to explore these interactions and provide more robust, stage-specific insights. Other imitations include the cross-sectional design, which does not allow causal inference. The modest sample size and reliance on self-reported measures for physical activity and QoL may introduce bias [36]. Despite these limitations, the cross-sectional approach is valuable for identifying associations and generating hypotheses about how psychological, cognitive, and physical factors interact across disability levels in MS.

In participants with severe disability, the observed weaker associations between psychological distress and QoL may reflect a “ceiling effect,” where extreme physical limitations dominate daily functioning and overshadow the influence of emotional symptoms. However, this should be interpreted cautiously as a hypothesis rather than a definitive conclusion. Alternative explanations are also plausible. For example, a response shift may occur, whereby patients adjust their internal standards or perceptions of QoL over time, leading to smaller apparent differences across psychological distress levels. Similarly, adaptation to chronic limitations or measurement insensitivity of the instruments in capturing subtle psychosocial variations in severely disabled patients may contribute to these findings. These considerations highlight the need for longitudinal studies and more sensitive assessment tools to better understand the interplay of psychological and functional factors in advanced MS.

Although several MusiQoL subdomains showed statistically significant differences across disability groups, the global MusiQoL score varied only modestly. This indicates that, while specific aspects of daily functioning, psychological well-being, and social relationships are affected, the overall quality of life as perceived by patients may remain relatively stable. Therefore, the clinical relevance of these subdomain differences should be interpreted cautiously. This emphasizes the importance of considering both individual subdomains and the global score when assessing patient-centered outcomes in MS.

### 4.2. Implications

Our findings support the use of disability-stage-informed, multidisciplinary care in MS, based on observed associations between psychological distress, cognitive function, physical activity, and QoL [37]. In individuals with mild-to-moderate disability, routine screening for anxiety, stress, and depression, alongside cognitive assessment and physical activity counseling, may help identify patients at risk of poorer functioning and well-being [38]. In patients with severe disability, strategies emphasizing physical support and adaptive functioning may be more impactful, as psychological distress appears less influential [6]. In contrast, among those with severe disability, care strategies may benefit from prioritizing physical assistance, adaptive rehabilitation, and healthcare engagement, as psychological factors showed more limited associations with QoL outcomes. These insights can guide clinical management, rehabilitation planning, and the development of patient-centered care strategies that consider disability stage [39]. Future longitudinal studies are warranted to evaluate the causal relationships between these domains and to test integrated interventions across the spectrum of MS disability.

These results highlight the value of integrated, patient-centered management approaches tailored to disability severity. Future longitudinal and interventional studies are needed to clarify temporal relationships, assess treatment responsiveness across disability stages, and inform evidence-based, stage-specific rehabilitation programs.

## 5. Conclusions

In people with MS, psychological distress—including anxiety, stress, and depression—is associated with lower ADL, PWB, symptom burden, coping, and social functioning, particularly in those with mild-to-moderate disability. Cognitive function and physical activity are also linked to these outcomes, highlighting the multifactorial nature of QoL in MS. These results underscore the value of individualized, multidisciplinary approaches that address mental health, cognitive support, and physical activity, tailored to disability stage. Future longitudinal and interventional studies are needed to clarify temporal relationships and inform stage-specific strategies for optimizing QoL in MS populations.

## Figures and Tables

**Table 1:** Sociodemographic, psychological, and quality-of-life characteristics by disability severity in patients with multiple sclerosis.

Characteristics	Disability Severity			*p*-Value
	Mild N = 65	Moderate N = 69	Severe N = 15	
Age, mean ± SD	32.1 ± 5.7	34.6 ± 4.4	40.8 ± 4.8	<0.001 *
Educational level, n (%)				0.018 ^†^
High school	4 (6.2)	9 (13)	4 (26.7)	
University	61 (93.8)	60 (87)	11 (73.3)	
Gender, n (%)				0.251 ^†^
Male	35 (53.8)	43 (62.3)	11 (73.3)	
Female	30 (46.2)	26 (37.7)	4 (26.7)	
Anxiety, n (%)				0.022 ^†^
Normal	26 (40)	17 (24.6)	5 (33.3)	
Mild	15 (23.1)	31 (44.9)	2 (13.3)	
Moderate	15 (23.1)	15 (21.7)	8 (53.3)	
Severe	6 (9.2)	1 (1.4)	0 (0.0)	
Extremely severe	3 (4.6)	5 (7.2)	0 (0.0)	
Stress, n (%)				<0.001 ^†^
Normal	42 (64.6)	22 (31.9)	1 (6.7)	
Mild	9 (13.8)	9 (13)	4 (26.7)	
Moderate	13 (20)	34 (49.3)	5 (33.3)	
Severe	1 (1.5)	4 (5.8)	5 (33.3)	
Extremely severe	0 (0.0)	0 (0.0)	0 (0.0)	
Depression, n (%)				0.001 ^†^
Normal	36 (55.4)	24 (34.8)	2 (13.3)	
Mild	12 (18.5)	7 (10.1)	8 (53.3)	
Moderate	16 (24.6)	36 (52.2)	5 (33.3)	
Severe	1 (1.5)	1 (1.4)	0 (0.0)	
Extremely severe	0 (0.0)	1 (1.4)	0 (0.0)	

Abbreviations: SD, standard deviation. Note: Disability severity was categorized as mild (0–3), moderate (4–6), and severe (7 or higher) based on the PDDS. * One-way ANOVA for age. ^†^ Chi-square test for categorical variables (education, gender, anxiety, stress, depression). Significance set at *p* < 0.05.

**Table 2:** Associations of cognitive function and physical activity with quality of life and daily functioning in multiple sclerosis patients across disability levels.

	Cognitive Function			Physical Activity			
	Low Mean ± SD	Normal Mean ± SD	*p*-Value	Low or No Activity Mean ± SD	Moderate Activity Mean ± SD	High Activity Mean ± SD	*p*-Value
**Mild**							
ADL	69.5 ± 8.3	82.8 ± 9.2	0.003	84.5 ± 10.7	79.9 ± 10.6	81.5 ± 7.7	0.304
PWB	71 ± 11.9	77.2 ± 12.9	0.301	81.2 ± 12.1	78.3 ± 12.0	71.1 ± 12.9	0.029
SYM	74 ± 11.9	84.2 ± 11.8	0.068	82.2 ± 12.1	82.4 ± 13.1	85.4 ± 13.4	0.616
RF	38.7 ± 10.9	45.3 ± 13.0	0.272	47.0 ± 14.6	42.0 ± 10.9	45.8 ± 13.4	0.423
RFAM	25.4 ± 11.9	30.2 ± 11.9	0.380	28.7 ± 8.9	28.1 ± 10.6	32.7 ± 14.9	0.374
SSL	52.0 ± 16.4	48.2 ± 12.7	0.527	46.5 ± 13.1	48.7 ± 14.6	50.0 ± 11.1	0.683
COP	76.0 ± 5.9	80.9 ± 12.2	0.381	80.7 ± 9.4	78.2 ± 12.6	82.7 ± 13.1	0.457
REJ	78.0 ± 4.5	77.3 ± 10.9	0.893	83.0 ± 7.3	78.7 ± 10.1	70.9 ± 10.2	0.000
RHCS	48.0 ± 13.0	52.0 ± 16.2	0.594	62.5 ± 12.1	44.8 ± 16.7	49.1 ± 13.4	0.000
MusiQoL	59.2 ± 6.3	64.2 ± 5.5	0.052	66.3 ± 5.3	62.4 ± 5.5	63.2 ± 5.7	0.062
**Moderate**							
ADL	68.6 ± 10.3	77.72 ± 8.9	0.002	73.6 ± 13.8	77.9 ± 10.1	75.5 ± 5.6	0.369
PWB	67.31 ± 14.4	68.8 ± 14.9	0.738	72.19 ± 19.7	70.9 ± 14.3	63.6 ± 10.0	0.103
SYM	80.0 ± 12.9	82.3 ± 10.4	0.491	75.0 ± 14.0	83.9 ± 11.1	84.0 ± 5.6	0.013
RF	44.1 ± 7.9	48.9 ± 14.2	0.243	52.5 ± 17.4	42.9 ± 10.9	50.9 ± 11.2	0.025
RFAM	43.1 ± 19.6	36.1 ± 17.3	0.204	30.4 ± 13.3	43.6 ± 21.9	34.9 ± 12.8	0.041
SSL	60.0 ± 12.9	53.2 ± 11.9	0.073	50.0 ± 15.5	55.7 ± 12.9	56.0 ± 8.7	0.252
COP	82.0 ± 9.9	85.2 ± 13.4	0.424	81.7 ± 9.3	82.8 ± 16.6	88.5 ± 8.9	0.159
REJ	73.1 ± 9.5	71.9 ± 9.2	0.698	76.2 ± 7.2	71.1 ± 12.6	70.8 ± 4.0	0.129
RHCS	50.8 ± 13.8	54.8 ± 11.4	0.273	54.4 ± 15.0	54.6 ± 10.4	53.2 ± 11.8	0.904
MusiQoL	63.2 ± 4.7	64.3 ± 5.4	0.485	62.9 ± 5.4	64.8 ± 5.9	64.2 ± 4.3	0.504
**Severe**							
ADL	56.5 ± 10.7	59.2 ± 3.8	0.680	56.9 ± 10.4	57.5 ± 3.5	–	0.941
PWB	72.1 ± 10.3	70.0 ± 10.0	0.758	70.8 ± 9.9	77.5 ± 10.6	–	0.392
SYM	77.1 ± 8.4	71.7 ± 2.9	0.301	75.4 ± 8.0	80.0 ± 7.1	–	0.459
RF	45.5 ± 12.3	40.0 ± 0.0	0.461	44.1 ± 11.7	46.6 ± 9.4	–	0.777
RFAM	38.3 ± 13.7	35.5 ± 16.7	0.765	37.9 ± 14.8	36.6 ± 4.7	–	0.906
SSL	68.3 ± 11.9	50.0 ± 10.0	0.030	66.1 ± 13.9	55.0 ± 7.1	–	0.295
COP	77.2 ± 4.5	80.0 ± 6.7	0.392	78.4 ± 4.8	73.3 ± 0.0	–	0.170
REJ	78.3 ± 3.9	80.0 ± 0.00	0.484	78.5 ± 3.7	80.0 ± 0.0	–	0.584
RHCS	45.0 ± 9.1	53.3 ± 5.8	0.158	46.9 ± 9.5	45.0 ± 7.1	–	0.790
MusiQoL	62.0 ± 2.9	60.0 ± 3.6	0.317	61.7 ± 3.2	61.3 ± 3.1	–	0.875

Abbreviations: SD, standard deviation; ADL, activity of daily living; PWB, physical well-being; SYM, symptoms; RF, relationship with friends; RFAM, relationships with family; SSL, sentimental and sexual life; COP, coping; REJ, rejection; RHCS, relationship with healthcare system; MusiQoL, musiquality of life total score.

**Table 3:** Associations of anxiety, stress, and depression with quality of life and daily functioning among MS patients across disability levels.

Outcome	Disability	N	Anxiety, Mean ± SD (Normal/Mild/Moderate/Severe/Extremely Severe)	*p*-Value	Stress, Mean ± SD (Normal/Mild/Moderate/Severe/Extremely Severe)	*p*-Value	Depression, Mean ± SD (Normal/Mild/Moderate/Severe/Extremely Severe)	*p*-Value
ADL	Mild	65	85.2/77.2/85.0/75.0/73.3	0.006	85.2/75.1/76.1/77.5/–	0.002	85.3/79.3/76.1/77.5/–	0.009
	Moderate	69	85.4/75.8/68.3/72.5/69.0	0.000	77.5/73.9/76.9/65.0/–	0.098	78.1/71.1/76.5/55.0/62.5	0.046
	Severe	15	59.5/55.0/55.9/–/–	0.797	80.0/58.7/58.0/50.0/–	0.013	56.2/61.2/50.5/–/–	0.146
PWB	Mild	65	85.8/68.7/76.3/68.3/58.3	0.000	83.2/67.8/63.8/79.0/–	0.000	83.7/73.5/64.7/55.0/–	0.000
	Moderate	69	85.0/62.1/67.3/65.0/57.0	0.000	82.0/73.3/60.0/56.2/–	0.000	82.9/68.6/59.7/50.0/60.0	0.000
	Severe	15	74.0/72.5/70.0/–/–	0.799	85.0/67.5/65.0/79.0/–	0.033	62.5/73.7/72.0/–/–	0.385
SYM	Mild	65	89.0/82.3/82.3/68.3/75.0	0.001	85.2/78.9/81.1/75.0/–	0.368	86.7/86.2/74.4/75.0/–	0.003
	Moderate	69	88.5/83.4/77.3/65.0/67.0	0.000	83.9/79.4/82.9/67.5/–	0.032	83.5/77.9/82.4/65.0/70.0	0.269
	Severe	15	74.0/75.0/77.5/–/–	0.751	85.0/75.0/79.0/72.0/–	0.360	75.0/78.1/73.0/–/–	0.543
RF	Mild	65	38.9/47.6/48.4/47.8/57.8	0.028	42.5/50.4/47.2/60.0/–	0.190	41.1/48.3/49.6/60.0/–	0.056
	Moderate	69	36.4/50.3/53.8/40.0/57.3	0.000	45.1/39.3/51.2/56.7/–	0.032	42.2/50.5/51.1/46.7/60.0	0.105
	Severe	15	38.7/53.3/45.8/–/–	0.274	26.7/41.7/42.7/52.0/–	0.151	40.0/45.0/45.3/–/–	0.852
RFAM	Mild	65	27.9/35.1/28.9/26.7/31.1	0.389	28.1/26.7/36.9/40.0/–	0.070	26.7/32.8/34.2/40.0/–	0.100
	Moderate	69	23.9/47.3/34.2/20.0/34.7	0.000	26.4/28.2/47.1/36.7/–	0.000	24.4/32.4/46.7/40.0/46.7	0.000
	Severe	15	31.9/36.6/41.7/–/–	0.495	20.0/36.6/32.0/48.0/–	0.137	20.0/39.2/42.7/–/–	0.128
SSL	Mild	65	50.0/52.0/45.3/43.3/43.3	0.443	47.6/42.2/55.4/50.0/–	0.107	46.9/52.5/48.7/50.0/–	0.648
	Moderate	69	51.2/59.0/51.3/40.0/50.0	0.070	50.0/52.2/58.5/50.0/–	0.056	50.4/52.9/58.1/50.0/40.0	0.118
	Severe	15	58.0/55.0/71.2/–/–	0.124	60.0/50.0/66.0/76.0/–	0.016	55.0/61.2/74.0/–/–	0.141
COP	Mild	65	80.0/86.2/81.8/71.1/68.9	0.030	79.7/79.9/84.6/66.7/–	0.380	80.5/84.4/78.3/66.7/–	0.367
	Moderate	69	79.2/90.7/82.6/73.3/73.3	0.003	78.2/77.0/92.1/73.3/–	0.000	78.0/80.9/90.5/80.0/60.0	0.000
	Severe	15	78.7/76.6/77.5/–/–	0.877	80.0/78.3/77.3/77.3/–	0.962	83.3/77.5/75.9/–/–	0.193
REJ	Mild	65	79.2/80.0/80.0/76.7/80.0	0.136	80.0/76.7/69.2/80.0/–	0.011	81.4/71.7/72.5/60.0/–	0.004
	Moderate	69	71.8/71.3/72.7/90.0/48.0	0.374	74.1/72.2/70.3/52.5/–	0.300	74.6/71.4/70.8/70.0/70.0	0.646
	Severe	15	80.0/80.0/77.5/–/–	0.420	80.0/80.0/78.0/78.0/–	0.821	75.0/80.0/78.0/–/–	0.178
RHCS	Mild	65	45.3/50.0/62.0/51.7/56.7	0.026	52.1/45.6/53.8/60.0/–	0.610	51.4/52.5/51.2/60.0/–	0.957
	Moderate	69	50.0/57.1/54.0/60.0/48.0	0.239	50.0/50.0/57.3/52.5/–	0.154	50.8/51.4/57.8/30.0/40.0	0.021
	Severe	15	46.0/50.0/46.2/–/–	0.871	30.0/55.0/48.0/42.0/–	0.016	50.0/47.5/44.0/–/–	0.708
MusiQoL	Mild	65	64.6/61.5/65.6/58.8/60.5	0.093	64.9/60.4/63.1/62.7/–	0.172	64.9/64.6/61.1/62.7/–	0.154
	Moderate	69	63.5/66.3/62.4/58.4/58.9	0.006	63.1/60.6/66.3/59.5/–	0.002	62.8/61.9/65.9/54.1/56.6	0.008
	Severe	15	60.1/61.5/62.6/–/–	0.378	60.7/60.3/60.7/63.8/–	0.301	57.5/62.6/61.7/–/–	0.097

Abbreviations: SD, standard deviation; ADL, activity of daily living; PWB, physical well-being; SYM, symptoms; RF, relationship with friends; RFAM, relationships with family; SSL, sentimental and sexual life; COP, coping; REJ, rejection; RHCS, relationship with healthcare system; MusiQoL, multiple sclerosis international quality of life.

## Data Availability

Data used in this study are available upon request from the corresponding author.

## Abbreviations

The following abbreviations are used in this manuscript:

MS	Multiple Sclerosis
QoL	Quality of Life
PDDS	Patient-Determined Disease Steps
DASS-21	Depression, Anxiety, Stress Scale-21
IPAQ	International Physical Activity Questionnaire
MusiQoL	Multiple Sclerosis International Quality of Life
RRMS	Relapsing–Remitting Multiple Sclerosis
MoCA	Montreal Cognitive Assessment
ANOVA	Analysis of Variance
SD	Standard Deviation
ADL	Activity of Daily Living
PWB	Psychological Well-Being
SYM	Symptoms
RF	Relationship with Friends
RFAM	Relationship with Friends and Family
SSL	Social Support
REJ	Rejection
RHCS	Scores For Healthcare Relationship
